# Robot-assisted Reduction Pyeloplasty with 3D Image Navigation for Adult Giant Hydronephrosis: Technique and Clinical Outcomes

**DOI:** 10.1590/S1677-5538.IBJU.2025.0525

**Published:** 2025-12-20

**Authors:** Hao Dong, Pan Song, Zhihua Li, Xiang Wang, Kunlin Yang, Xuesong Li

**Affiliations:** 1 Peking University First Hospital Department of Urology Beijing China Department of Urology, Peking University First Hospital, Beijing, China; 2 Peking University Institution of Urology Beijing China Institution of Urology, Peking University, Beijing, China; 3 National Urological Cancer Center Beijing China National Urological Cancer Center, Beijing, China

**Keywords:** Imaging, Three-Dimensional, Robotic Surgical Procedures, Hydronephrosis

## Abstract

**Purpose:**

To describe the surgical technique and evaluate the clinical outcomes of robot-assisted reduction pyeloplasty for adult giant hydronephrosis (GH) secondary to ureteropelvic junction obstruction (UPJO).

**Materials and Methods:**

Between May 2019 and August 2024, 18 adult patients with GH caused by UPJO underwent robot-assisted laparoscopic reduction pyeloplasty. Patients’ characteristics, perioperative variables, and clinical outcomes were prospectively recorded. Three-dimensional (3D) reconstructions generated from CTU were used for preoperative planning and intraoperative navigation. The surgical technique was described, and outcomes were assessed.

**Results:**

All procedures were completed successfully with no conversions to open surgery. The median (range) operative time was 153 (77-241) minutes, with a median (range) estimated blood loss of 20 (10-100) mL. No intraoperative complications were observed. During a median (range) follow-up of 10 (6-40) months, all patients achieved complete symptomatic relief and significant reduction in hydronephrosis. Renal parenchymal thickness improved significantly after surgery (11.9 ± 3 mm vs 9.2 ± 4.4 mm, P=0.0207). Split renal function [38.7 (15.4-48.7) vs 25.7 (3.6-53.5), P=0.0131] showed significant improvement after surgery, which was consistent in patients in poorly functioning kidney subgroup [26.0 (19.2-24.6) vs 21.9 (11.6-24.6), P=0.0273].

**Conclusion:**

Our results show that robot-assisted reduction pyeloplasty is a safe and effective option for managing GH, facilitating significant improvement in renal functional outcomes, even in patients with borderline renal function.

## INTRODUCTION

Giant hydronephrosis (GH) is an uncommon but clinically significant urological condition, predominantly reported in children and rarely observed in adults (1, 2). It is typically defined as the accumulation of more than 1000 mL of fluid within the renal collecting system. Radiographically, it is characterized by a hydronephrotic kidney that crosses the midline or extends more than the height of five vertebral bodies (2). The most common underlying cause is ureteropelvic junction obstruction (UPJO), followed by urolithiasis, distal ureteral stricture, and tumors (1).

Reconstructive surgery is considered the optimal treatment for GH, with the goals of relieving obstruction and preserving renal function. However, the severe anatomical distortion and mass effect of GH make the surgical reconstruction in these patients particularly challenging. In addition to the anatomical obstruction caused by UPJO, this condition also exhibits a functional obstruction arising from increased non-functional intrarenal space, which could cause urinary stasis and thereby increase the risk of urolithiasis and infection (3, 4). Reduction pyeloplasty, derived from traditional dismembered pyeloplasty, is designed to excise the redundant pelvis and restore a funnel-shaped configuration (5). This non-kidney-invasive approach could effectively reduce intrarenal dead space and optimize urinary drainage.

In the era of minimally invasive surgery, laparoscopic pyeloplasty has gradually supplanted open pyeloplasty because of its minimal invasiveness and shorter recovery (6). However, the limitations of laparoscopic surgery, including two-dimensional (2D) visualization and restricted instrument dexterity, are amplified in complex UPJO reconstructions, especially in GH (7, 8). Moreover, reduction pyeloplasty involving extensive excision of the redundant renal pelvis, requires more and precise intracorporeal suturing, which increases the risk of urine leakage (5, 9). Recently, robot-assisted surgery has been widely adopted for complex urinary tract reconstruction, owing to its unique advantages of magnified three-dimensional (3D) vision and better intracorporeal suturing (8,9). Nevertheless, its application in the management of GH secondary to UPJO remains scarcely reported, especially in adults (9–11). We hypothesize that robot-assisted reduction pyeloplasty, assisted by three-dimensional (3D) image navigation, may improve surgical precision and facilitate renal function preservation in these complex cases. This study describes the surgical technique and clinical outcomes of robot-assisted reduction pyeloplasty for adult GH, aiming to provide a safe, feasible, and minimally invasive alternative for this rare but challenging condition.

## MATERIALS AND METHODS

### Study Population

Between May 2019 and August 2024, eighteen patients diagnosed with GH secondary to UPJO underwent robot-assisted reduction pyeloplasty, performed by an experienced surgeon. Patients’ characteristics, perioperative data, and clinical outcomes were prospectively recorded in the Reconstruction of Urinary Tract: Technology, Epidemiology and Result (RECUTTER) database. All procedures were conducted following the standards of the Ethics Committee of Peking University First Hospital (No. 2023-602) and the Declaration of Helsinki (as revised in 2013).

Inclusion criteria were as follows: (1) adult patients diagnosed with GH secondary to UPJO who underwent robot-assisted reduction pyeloplasty; (2) patients with preoperative CTU available for 3D reconstruction. Exclusion criteria were as follows: (1) patients with incomplete data or follow-up; (2) patients with GH caused by other etiologies.

**Figure 1:**
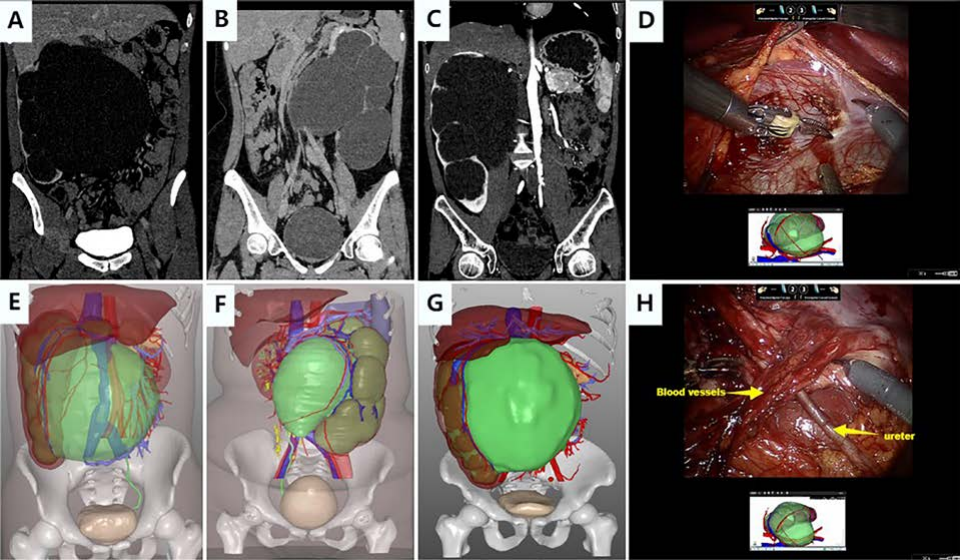
Representative CTU images of GH and the application of 3D reconstruction images in preoperative planning and intraoperative navigation.

The diagnosis of GH was based on the computed tomography urography (CTU) with 3D reconstructions (Figure-1). Giant hydronephrosis was defined as a hydronephrotic volume exceeding 1000 mL. Radiologically, it was characterized by a dilated kidney that crossed the midline or extended beyond the height of five vertebral bodies (1, 2). Ultrasonography and CTU were routinely conducted in all patients. Diuretic renography was employed to evaluate affected renal function. Poorly functioning kidney (PFK) was defined as split renal function (SRF) ≤ 30% (12). Ureteral stent placement or percutaneous nephrostomy (PCN) was performed in some patients to alleviate hydronephrosis and preserve renal function. In patients who underwent PCN, daily nephrostomy drainage was recorded to assess affected renal function. 3D image generated by CTU was utilized for preoperative planning and intraoperative navigation, enabling improved anatomical recognition and reducing the risk of iatrogenic injury (13).

(A-C) CTU images in three representative patients with GH. A) Marked dilatation of the renal collecting system with absence of excretory-phase opacification, indicating severe urinary obstruction; B) Mass effect of GH with compression of major abdominal vessels and adjacent organs; C) Hydronephrotic kidney with markedly thinned parenchyma and preserved arterial-phase cortical enhancement, suggesting residual renal function.

(E-G) 3D reconstruction images for perioperative planning. E) Visualize GH (green) occupying most of the abdomen; F) Identify surrounding vessels (arteries in red and veins in blue); G) Clarify spatial relationships between GH and adjacent organs (liver in brown and pancreas in yellow). (D and H) The application of intraoperative 3D image navigation by the surgeon's cognitive fusion. D) Guide dissection to avoid iatrogenic injury; H) Identify critical structures in distorted anatomy.

**CTU = computed tomography urography; GH = giant hydronephrosis; 3D = three-dimensional**

## SURGICAL TECHNIQUE

**Figure 2:**
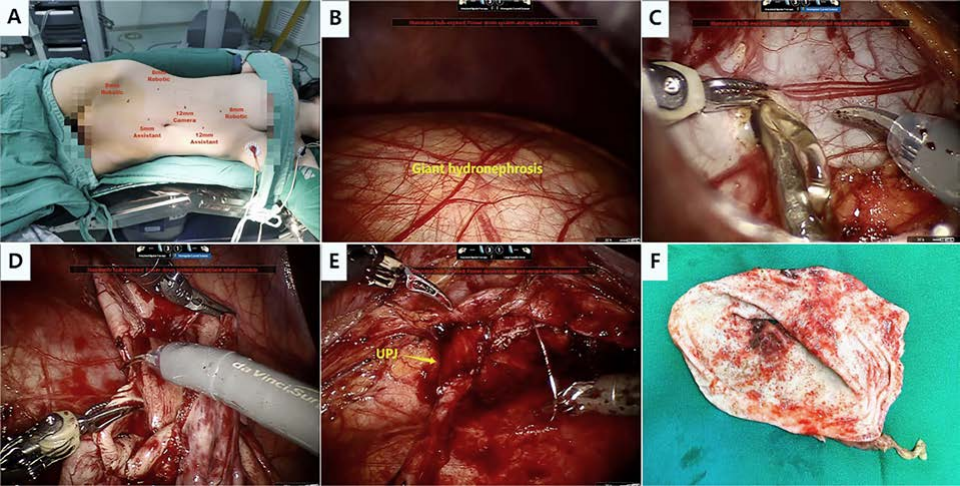
Intraoperative images of robot-assisted reduction pyeloplasty.

Patient positioning, port placement and surgical images are shown in Figure-2. Following induction of general anesthesia and tracheal intubation, transurethral retrograde double-J stent placement was performed in cases without preoperative drainage of hydronephrosis. The patient was then positioned laterally (45-60°) with the affected side facing upward. Four robotic ports (one 12 mm optical trocar and three 8 mm robotic trocars) and two assistant ports (one 5 mm trocar and one 12 mm trocar) were typically utilized. All procedures were performed using a transperitoneal approach. After incising the posterior peritoneum along the paracolic gutter, the colon was mobilized medially. Due to the severely enlarged kidney occupying most of the operative field, dissection and exposure of the ureteropelvic junction became extremely difficult. To alleviate the mass effect of GH, the markedly dilated renal pelvis was incised, and a large volume of intrarenal urine was aspirated. Meanwhile, adjacent organs and vessels were carefully protected to avoid iatrogenic injury. 3D images were utilized for intraoperative navigation by the surgeon's cognitive fusion during dissection (Figure-1).

A) Patient position and port placement for robot-assisted laparoscopic reduction pyeloplasty; B) Marked mass effect of giant hydronephrosis; C) Decompression of the dilated collecting system to enlarge the operative workspace; D) Excision of the redundant pelvis; E) Funnel-shaped ureteropelvic junction configuration following reconstruction; F) Resected renal pelvis tissue and stenotic ureteric segment.

Following adequate identification of the renal pelvis and ureter, an oblique incision was made in the renal pelvis. Redundant renal pelvic tissue was excised to reduce the pelvic size and improve drainage efficiency. The ureteral stricture was subsequently incised longitudinally until healthy ureteral tissue was encountered. Pyeloplasty was then performed, followed by continuous tension-free suturing. The first stitch was placed between the lowest corner of the renal pelvis and the ureter to prevent torsion and to serve as a landmark for the subsequent anastomosis. The posterior-wall anastomosis was completed first. In cases without a preexisting double-J stent, stent insertion was performed using a flexible guidewire before anastomosis of the anterior wall. Finally, the open renal pelvis was sutured. The ureteropelvic anastomosis was configured into a funnel shape following hydrodynamic principles to optimize renal pelvic drainage. In cases with concomitant renal calculi, stones were removed by forceps under direct vision.

### Postoperative treatment and follow-up

The Foley catheter was removed on postoperative Day 7. The double-J stent was removed 2 months after surgery by cystoscopy. At 3 months postoperatively, the modified Whitaker test was performed in patients with a nephrostomy tube to evaluate the feasibility of tube removal (14). Postoperative follow-up was conducted at three-month intervals during the first year and at six-month intervals thereafter, up to the second year. Postoperative complications were defined and graded by the Clavien-Dindo (CD) classification (15). Hydronephrosis was assessed using renal ultrasonography, CTU, and magnetic resonance urography. Changes in renal morphology were assessed using renal parenchymal thickness (RPT). All RPT measurements were performed by senior urological ultrasonography doctors with more than 5 years of experience, following standardized urological ultrasound protocols. For each patient, preoperative and postoperative measurements were performed by the same doctor to control inter-observer variability.

Renal function was evaluated by glomerular filtration rate (GFR) and SRF of diuretic renography. Renal function outcomes were categorized as improvement, stability and deterioration. Based on biological variation studies in both healthy and chronic kidney disease populations, the physiological fluctuation of estimated glomerular filtration rate is approximately 12.5%-16.5% (16, 17). To minimize the impact of inherent variability on our results, we therefore defined a relative change of 20% in renal functional parameters as the threshold for a meaningful change. Improvement was defined as an increase of ≥20% in SRF for non-solitary kidneys or in GFR for solitary kidneys at the last follow-up relative to baseline. Deterioration was defined as a ≥20% decline in SRF for non-solitary kidneys or in GFR for solitary kidneys at the last follow-up relative to baseline. Stability was defined as changes within ± 20% of baseline values.

## Statistical Analysis

Categorical variables were expressed as frequency (percentage). The distribution of continuous variables was first assessed using the Shapiro-Wilk test. Continuous variables with a normal distribution were presented as mean ± standard deviation (SD), while those not following a normal distribution were reported as median (range). For paired comparisons of preoperative and postoperative parameters, the distribution of paired differences was assessed using the Shapiro-Wilk test. If the paired differences were normally distributed, data were compared using the paired t-test. If the paired differences were not normally distributed, data were compared using the Wilcoxon signed-rank test. All statistical analyses were performed using SPSS software (version 27.0), and a P-value < 0.05 was considered statistically significant.

## RESULTS

As shown in Table-1, 18 patients diagnosed with GH were included, comprising 11 men and 7 women. The mean age was 26.8 ± 10.0 years. All cases of GH were attributed to UPJO. The left side was affected in 9 (50.0%) patients. In terms of clinical presentation, 12 (66.7%) patients experienced flank pain, 1 (5.6%) patient presented with an abdominal mass, and 5 (11.1%) patients were asymptomatic. 17 (94.4%) patients had primary UPJO, 1 (5.6%) patient had a history of failed endoscopic ureteral balloon dilatation, and no patient had a history of prior pyeloplasty. 9 (56.2%) patients were diagnosed with PFK. For preoperative drainage, 3 (16.6%) cases had double-J stent placement and 6 (33.3%) had PCN. For patients with PCN, nephrostomy output was recorded. The median (range) nephrostomy output was 2000 (700-3000) mL. All patients underwent robot-assisted reduction pyeloplasty. All procedures were completed successfully without conversion to open surgery. The median (range) operative time was 153 (77-241) minutes, and the median (range) estimated blood loss was 20 (10-100) mL. No perioperative complications were recorded.

**Table 1:** Patients’ characteristics and perioperative data.

Variable		Results
Number of patients, n		18
**Gender, n (%)**		
	Male	11 (61.1)
	Female	7 (38.9)
Age (years), mean ± SD		26.8 ± 10.0
BMI (kg/m^2^), mean ± SD		22.0 ± 3.2
**Affected side, n (%)**		
	Left	9 (50.0)
	Right	9 (50.0)
**Clinical presentation, n (%)**		
	Flank pain	12 (66.7)
	Abdominal mass	1 (5.6)
	No symptom	5 (27.8)
Solitary kidney, n (%)		2 (11.1)
**SRF group, n (%)**		
	> 30%	7 (43.8)
	≤ 30%	9 (56.2)
History of endoscopic dilation, n (%)		1 (5.6)
History of ureteral reconstruction, n (%)		0 (0.0)
Concomitant urolithiasis (n%)		2 (11.1)
Preoperative DJ stent indwelling, n (%)		3 (16.6)
**Preoperative PCN, n (%)**		6 (33.3)
	PCN output (mL), median (range)	2000 (700-3000)
Operative time (min), median (range)		153 (77-241)
Conversion to open surgery		0 (0/18)
Estimated blood loss (mL), median (range)		20 (10-100)
Postoperative hospitalization (day), median (range)		4 (4-6)

BMI = body mass index; SRF = split renal function; PCN = percutaneous nephrostomy; SD = standard deviation

**Figure 3:**
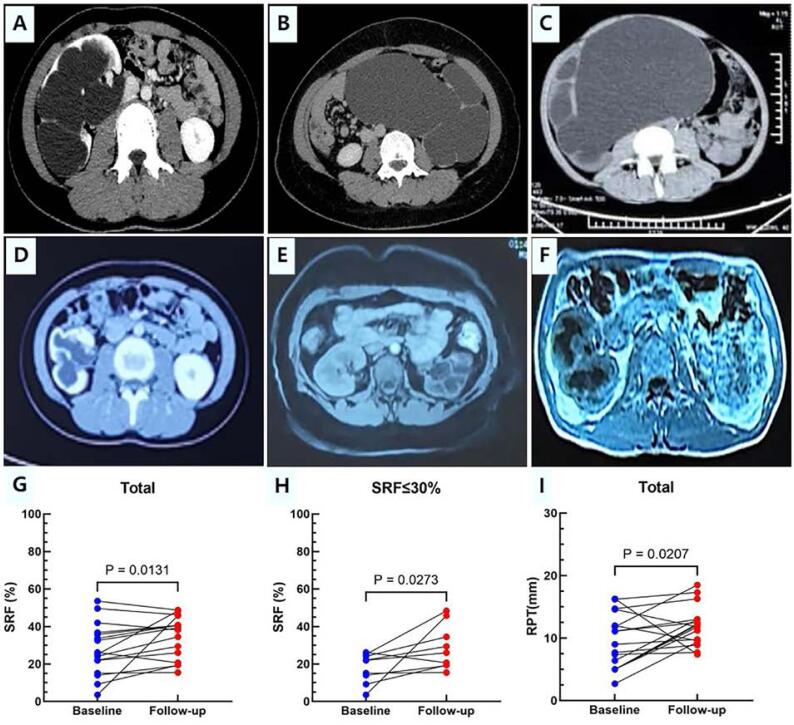
Renal function and morphological outcomes.

The clinical outcomes are shown in Table-2 and Figure-3. The median (range) follow-up period was 10 (6-40) months. Postoperative imaging demonstrated a substantial reduction in hydronephrosis. We compared the RPT before surgery and at the last follow-up. It revealed a significant improvement in RPT (11.9 ± 3.0 mm vs 9.2 ± 4.4 mm, P = 0.0207) after surgery (Table-2 and Figure-3I). All patients experienced relief of clinical symptoms. During follow-up, a 4.7 mm renal calculus developed in 1 patient, who remained asymptomatic and was managed conservatively. No other major long-term complications, including urinary tract infection or recurrent obstruction, were observed.

**Table 2:** The clinical outcomes of patients.

Variable		Results
Number of the total patients, n		18
Number of non-SK		16
Number of SK		2
Follow up time (months), median (range)		10 (6-40)
Preoperative Scr (μmol/L), mean ± SD		85.7 ± 17.6
Follow-up Scr (μmol/L), mean ± SD		82.9 ± 13.6
Preoperative SRF for non-SK (%), median (range)		25.7 (3.6-53.5)
Follow-up SRF for non-SK (%), median (range)		38.7 (15.4-48.7)
Preoperative GFR for SK (mL/min/1.73 m^2^), mean ± SD		53.5 ± 2.1
Follow-up GFR for SK (mL/min/1.73 m^2^), mean ± SD		79.3 ± 2.4
Preoperative RPT (mm), mean ± SD		9.2 ± 4.4
Follow-up RPT (mm), mean ± SD		11.9 ± 3.0
Symptom relief, n (%)		18 (100)
**Renal function, n (%)**		
	Improvement	10 (55.6)
	Stability	7 (38.9)
	Deterioration	1 (5.6)
Hydronephrosis improvement, n (%)		18 (100%)
Long term complication, n (%)		1 (5.6)

Scr = serum creatinine; SRF = split renal function; GFR = glomerular filtration rate; SK = solitary kidney; RPT = renal parenchymal thickness; SD = standard deviation

(A-C) Baseline images for GH. A) Cystic dilatation of the collecting system with parenchymal thinning; B) Mass effect of GH compressing adjacent organs; C: Severe hydronephrosis crossing the midline with thinning parenchyma.

(D-F) Significant radiographic improvement after surgery. D) Marked reduction in hydronephrosis with parenchymal thickening; E) Substantial resolution of mass effect. F) Marked reduction in dilatation of renal pelvis.

G) Marked reduction in dilatation of renal pelvis. G) The change of SRF in 16 non-solitary kidney patients after surgery. The SRF was compared with a paired-sample t-test; H) The change of SRF in patients with PFK (n=9) after surgery. The SRF was compared with a paired- sample t-test; I) The change of RPT in total patients (n=18). The RPT was compared with a paired-sample t-test.

**GH = giant hydronephrosis; SRF = split renal function; PFK = poorly functioning kidney; RPT = renal parenchymal thickness**

For renal function outcomes, the last follow-up SRF [38.7 (15.4-48.7) vs 25.7 (3.6-53.5), P = 0.0131] for 16 non-solitary kidneys showed significant improvements compared to preoperative values, which was consistent in patients with PFK [26.0 (19.2-24.6) vs 21.9 (11.6-24.6), P = 0.0273]. In two patients with a solitary kidney, the mean postoperative increase in GFR was 25.8 mL/min/m2. In the overall cohort, renal function improved in 10 (55.6%) patients, remained stable in 7 (38.9%), and deteriorated in 1 (5.6%). In the patient with worsening renal function, SRF declined from 26.2% to 20.6%, representing a 21% decrease relative to baseline, and remained stable at this reduced level during follow-up. Despite this decrease, there was no evidence of recurrent obstruction, urinary tract infection, or other major postoperative complications, and no additional intervention was required.

## DISCUSSION

GH is a rare condition defined as hydronephrosis containing fluid more than 1000 mL (2). The etiology in approximately 80% of cases is UPJO (18). GH progresses slowly and insidiously, and flank or abdominal discomfort may be the only symptom. Such subtle signs are easily overlooked by patients, potentially resulting in the development of a non-functional kidney (19).

To date, there are no established consensus guidelines for the surgical management of GH. In clinical practice, the decision between nephrectomy and kidney-sparing surgery is primarily determined by the function of the affected kidney (19). Nephrectomy is generally recommended when SRF is poor (20). However, available methods of evaluating SRF in patients with GH have inherent limitations. Diuretic renography is the standard tool for SRF measurement, but increased intrarenal pressure in GH may impair radionuclide uptake, resulting in an underestimation of the true SRF, especially in young adults (21, 22). Renal parenchymal thickness (RPT) may reflect residual function, but severe distortion of renal anatomy caused by hydronephrosis and the operator-dependent ultrasound measurements may produce inconsistent results (23). Our results demonstrated that significant postoperative improvements in both SRF and RPT, providing preliminary evidence for the feasibility and benefits of kidney-sparing in GH, even among patients with borderline renal function. Although one patient experienced a postoperative functional decline, there was no evidence of restenosis or other complications. The preoperative symptoms resolved completely, and renal function remained stable at this reduced level throughout follow-up. Therefore, we considered that this patient still attained a clear clinical benefit from surgery.

Given the potential for renal preservation and its clinical benefits, the kidney-sparing surgery is recommended in patients with GH, especially in younger individuals. However, reconstruction in GH remains technically challenging, which demands meticulous dissection and intracorporeal suturing, and knotting within a confined, distorted operative field (9). The robotic technique offers several advantages, such as 3D visualization, greater dexterity, and precise suturing, thereby facilitating complex reconstruction such as GH (8, 9, 24). However, existing studies on robot-assisted pyeloplasty for GH have limited generalizability due to small sample sizes (9–11). In the present study, robot-assisted reduction pyeloplasty with 3D image navigation was performed in 18 patients with GH. Perioperative and follow-up outcomes demonstrated that favorable results, including minimal blood loss, shorter hospital stay, and fewer complications, were achieved.

Optimal outcomes in such complex cases depend not only on advanced surgical technique but also on meticulous preoperative planning and intraoperative navigation with the assistance of 3D reconstruction based on CTU. During preoperative planning, 3D image clearly delineated the anatomy of the hydronephrotic kidney and adjacent vasculature (13, 25). Furthermore, intraoperative 3D image navigation by the surgeon's cognitive fusion was used to achieve more precise identification and dissection within the distorted anatomy, thereby potentially reducing the risk of iatrogenic injury. However, the generation of patient-specific 3D models is time-consuming, costly, and highly dependent on advanced radiology platforms and experienced operators. As a result, this technique has not been wildly adopted for routine clinical use and is currently more suitable for complex cases.

The mass effect caused by GH markedly interferes with precise dissection and adequate exposure of the ureteric stricture. Based on our experience, several strategies can be employed to mitigate these constraints on intracorporeal manipulation. First, preoperative decompression of hydronephrosis is essential. In patients without prior drainage, retrograde transurethral placement of a double-J stent is performed at the outset of surgery to achieve preliminary decompression. Secondly, a transperitoneal approach is adopted to provide a broader operative field. Careful robotic port placement is essential to avoid iatrogenic injury to bowel loops, renal pedicle vessels, and other vital structures, with the initial port inserted under direct vision when necessary. Thirdly, the markedly dilated renal pelvis is incised to aspirate the intrarenal fluid, thereby enlarging the workspace and facilitating subsequent dissection and reconstruction.

In GH, the affected kidney exhibits an extremely dilated renal collecting system and a thinned renal cortex. Even after the anatomical obstruction has been surgically relieved, functional obstruction factors, including redundant intrarenal space and compromised peristaltic activity of the collecting system, may still persist, leading to urinary stasis and predisposing patients to urolithiasis and infection (3, 4). Various surgical techniques have been employed to address this type of functional obstruction, including nephroplication, ureterocalicostomy, and reduction pyeloplasty (2, 3, 9, 26).

Nephroplication is a complex, parenchyma-invasive procedure in which the upper and lower renal poles are sutured and folded toward the middle pole, thereby facilitating calyceal drainage (3). However, this technique carries potential risks, including renal hemorrhage and parenchymal volume loss, which are of particular concern in patients with PFK. In a recent report, a novel suture-free nephroplication was introduced using a four-dimensional printed biodegradable pouch to compress and fold the dilated kidney (10). Although initial outcomes appear promising, further studies are needed to validate this technique before it can be widely adopted in clinical practice. Ureterocalicostomy involves excision of the lower renal pole and direct anastomosis of the lower calyx to the ureter (26). It is a viable reconstruction alternative for patients with a severely compromised collecting system due to prior failed pyeloplasty, as well as for those with anatomical anomalies such as an intrarenal pelvis (27–29).

To maximize renal function preservation in patients with GH, we prefer to choose a non-kidney-invasive surgical approach. In our study, robot-assisted reduction pyeloplasty was undertaken, which involved routine excision of the UPJ stricture, supplemented by resection of the redundant dilated pelvis (5). This volume-reducing strategy can decrease non-functional intrapelvic space and restore a funnel-shaped configuration, thereby optimizing urinary drainage (5). However, owing to the extensive reduction and a lengthy suture line required, laparoscopic execution is technically demanding with a prolonged learning curve (9). Difficulty in intracorporeal suturing has been identified as a major cause of conversion from laparoscopic to open surgery (30). The robotic surgical technique, with its advantages of 3D magnified visualization and precise suture, appears to effectively overcome these challenges, resulting in a shorter anastomosis time and a lower complication rate (8, 9). In our study, these advantages translated into favorable outcomes. All robot-assisted procedures were completed successfully without intraoperative complications. The mean operative time was 153 minutes, with an acceptable estimated blood loss.

In summary, this study describes robot-assisted reduction pyeloplasty for managing GH. This non-parenchyma-invasive procedure could effectively address both anatomical and functional obstructions. In addition, we incorporated CTU-based 3D reconstruction into preoperative planning and intraoperative cognitive fusion navigation to minimize the risk of iatrogenic injury in this challenging condition. Beyond symptomatic relief, we report postoperative improvements in both renal functional and morphological parameters, providing important evidence for kidney-sparing strategies for GH. However, several limitations of this study should be acknowledged. Firstly, although it represents the largest published cohort of robotic reconstruction for adult GH, the sample size remains limited due to the rarity of this condition. Secondly, we could not perform a comparison of reduction pyeloplasty with other approaches. Thirdly, the relatively short follow-up duration may have limited the evaluation of long-term renal functional outcomes. Multicenter studies with larger cohorts and prolonged follow-up are required to further validate these findings. Despite these limitations, our study provides valuable insights into the management of this rare but technically challenging condition and further provides important evidence for kidney-sparing strategies for GH.

## CONCLUSIONS

Nephrectomy should be performed with greater caution in patients with a poorly functioning kidney caused by giant hydronephrosis, especially in younger individuals. Robot-assisted laparoscopic reduction pyeloplasty with 3D image navigation is a safe and effective technique for managing giant hydronephrosis secondary to UPJO in adults and it promotes renal preservation even in patients with borderline renal function. However, large sample, multicenter, and long-term studies are essential in the future.

## Data Availability

All data generated or analysed during this study are included in this published article

## ABBREVIATIONS

3D: = three dimensional
2D: = two-dimensional
GH: = giant hydronephrosis
UPJO: = ureteropelvic junction obstruction
CTU: = computed tomography urography
GFR: = glomerular filtration rate
SRF: = split renal function
PFK: = poorly functional kidney
PCN: = percutaneous nephrostomy
CD: = Clavien-Dindo
RPT: = renal parenchymal thickness
